# Plasma concentrations of D-dimer and outcome of in vitro fertilization

**DOI:** 10.1186/1757-2215-7-58

**Published:** 2014-05-22

**Authors:** Marcello Di Nisio, Ettore Porreca, Valeria Di Donato, Gian Mario Tiboni

**Affiliations:** 1 Department of Medical, Oral and Biotechnological Sciences, University “G. D’Annunzio” of Chieti-Pescara, Chieti, Italy; 2 Department of Vascular Medicine, Academic Medical Center, Amsterdam, Netherlands; 3 Department of Medicine and Aging, University “G. D’Annunzio” of Chieti-Pescara, Chieti, Italy

**Keywords:** In-vitro fertilization, Clinical pregnancy, D-dimer, Hypercoagulability

## Abstract

**Background:**

The activation of blood coagulation could contribute to the failure of in-vitro fertilization (IVF) techniques. The aim of this study was to assess the predictive value of D-dimer levels for pregnancy outcome in women undergoing IVF.

**Findings:**

A prospective study was performed in 105 women undergoing IVF. D-dimer was measured before and one week after the administration of recombinant human chorionic gonadotropin (r-hCG). The primary outcome of the study was clinical pregnancy. The mean age was 36 years (range 26 to 43 years). The main indications for IVF were infertility due to a tubaric (n = 21, 20%) or male factor (n = 37, 35%) and idiopathic infertility (n = 30, 29%) which altogether accounted for 84% of the total. Clinical pregnancy was achieved by 40/105 (38%) women of whom 32 (80%) delivered a live child. On the day of r-hCG administration, D-dimer concentrations were significantly higher in patients not achieving a clinical pregnancy (141 ng/dL vs. 115 ng/dL, p = 0.035) which remained statistically significant after correction for age and indications for IVF in multivariable analysis (p = 0.032). One week after r-hCG, the levels of D-dimer were significantly increased both in women with and without a clinical pregnancy with no differences between the groups (748 ng/dL vs. 767 ng/dL, p = 0.88).

**Conclusions:**

D-dimer concentrations seem to predict a higher risk of pregnancy failure in women undergoing IVF. If confirmed in future prospective studies, D-dimer could help identifying a group of patients who could benefit from prophylaxis to increase the pregnancy success rate.

## Background

The average pregnancy rate after in-vitro fertilization (IVF) remains as low as 30% [1]. One of the possible mechanisms behind the high failure rate is the unsuccessful implantation or placentation due to hypercoagulability causing thrombosis of maternal vessels with reduced perfusion of the intervillous space and placentation failure [2]. A number of studies evaluated the causal relationship between states of hypercoagulability and outcomes of IVF reporting conflicting findings, as summarised in a recent systematic review of the literature [3].

The hormonal milieu resulting from the use of contraceptive pills or hormone replacement therapy has been clearly associated with hypercoagulability [4,5]. By contrast, only few studies examined the impact on haemostatic parameters of supra-physiological oestrogen levels as seen during IVF [6]. Although the interpretation of the data remains difficult due to the relatively small size of the studies and the heterogeneity of IVF protocols, the available evidence suggests that ovarian stimulation during IVF is associated with increased concentrations of coagulation factors and impairment of endogenous anticoagulants. All these haemostatic changes seem amplified in cases of excessive ovarian response as it occurs in the ovarian hyperstimulation syndrome (OHSS) [7]. Interestingly, preliminary observations suggested that haemostatic markers such as D-dimer are associated with an unfavourable pregnancy outcome in women with OHSS [7].

The aim of this prospective study was to evaluate the association between plasma D-dimer levels and IVF outcome.

## Methods

### Patients

Women attending the Assisted Reproduction Unit of the Ortona General Hospital and undergoing IVF from January 2011 to December 2012 were eligible for the study. Indications for IVF treatment included anovulation, endometriosis, tubal factor, male factor, mixed factor and unexplained infertility. Exclusion criteria were the ongoing use of anticoagulants at prophylactic or therapeutic doses or unwillingness to provide consent for the participation in the study. High responders that were coasted to prevent OHSS were also excluded. The study was conducted in compliance with the Helsinki Declaration and informed consent was obtained from all participants.

### Ovarian stimulation protocol

Gonadotropin Releasing Hormone (GnRH) agonist or antagonist were used to prevent premature luteinizing hormone (LH) surges. Ovarian stimulation was performed using daily subcutaneous injections of recombinant follicle-stimulating hormone (r-FSH; Puregon, Merk Sharpe and Dome, Germany; Gonal F, Merk Serono, Switzerland) at doses ranging from 75 IU to 450 IU depending on the woman’s age, the antral follicle count (AFC), and the basal (day 3) FSH circulating level. Controlled ovarian stimulation was started on day 3 of full menstrual flow if pituitary suppression was complete, as shown by absence of ovarian cysts, serum estradiol < 50 pg/ml and endometrial thickness < 3 mm. Gonadotropin dosage was adjusted according to the individual response. Recombinant human chorionic gonadotropin (r-hCG; Ovitrelle, Merk-Serono, Switzerland ) was administered when at least three leading follicles reached 18 mm. Oocyte pick-up was scheduled 36 hours after r-hCG injection. All the collected oocytes were denuded and their nuclear maturation stage determined. All the in-vitro fertilizations were performed by intracytoplasmic sperm injection (ICSI). Embryo transfer was performed 72–76 hours after ovum pick-up. A luteal phase support of 600 mg/day micronized intravaginal progesterone (Progefik, Effikitalia, Italy) was started the day after oocyte retrieval and maintained until a pregnancy test was performed, or until the seventh week’s gestation if the patient tested positive. Pregnancy test was performed by dosing serum βhCG levels, 14 days after embryo transfer. Clinical pregnancy was defined as the presence of a gestational sac with accompanying fetal heart beat as observed by ultrasound at seven weeks gestation.

#### *Blood collection*

Venous blood was collected from an antecubital vein without venous stasis using a 19-gauge butterfly needle. Blood withdrawal was planned 48 hours before the administration of r-hCG (maximal estrogens concentration) and between eight to ten days afterwards. Blood samples for D-dimer test were collected into tubes containing a mixture of sodium citrate, centrifuged for 15 minutes at 3,000 × g, and stored at -80°C until further analysis. D-dimer concentrations were measured by a latex quantitative assay (D-dimer Plus assay, Dade Behring, Marburg, Germany) with a cut-off value of 200 ng/mL for an abnormal test result according to the manufacturer indications.

#### *Statistical analysis*

Information on baseline characteristics, risk factors, and treatments were analyzed by means of descriptive statistics. Variables with a non-parametric distribution were presented as medians (range) and those with a parametric distribution as means (± standard deviation). Differences between women achieving a clinical pregnancy and women with a pregnancy failure were tested with the Mann–Whitney U test or the T-test, as appropriate. Variables with a statistical significance corresponding to a p < 0.15 in univariate analysis were assessed in multivariate logistic regression analysis. D-dimer levels were analysed as a continuous variable and according to the cut-off of the assay for positivity (200 ng/dL) comparing values above and below the cut-off. All statistical analyses were performed using SPSS software (version 20; SPSS Inc, Chicago, Illinois).

## Results

The clinical characteristics of the study population are shown in Table 1. The mean age was 36 years (26 to 43 years). In over two thirds of the cases infertility was idiopathic or the consequence of a male or tubal factor. Overall, 5 (4.8%) of 105 women achieved a biochemical pregnancy, and 40 (38%) obtained a clinical pregnancy of whom 32 (80%) delivered a live child which was pre-term (before 37 weeks gestation) in 23 (72%) (Table 2). Twin pregnancy occurred in 9/40 (22%) women, and in one case pregnancy was complicated by intra uterine growth restriction. Seven women aborted before the 12th week gestation (17%) and one experienced an extra uterine pregnancy. There were no thromboembolic complications during the study and pharmacological thromboprophylaxis was not administered to any patient during IVF.

**Table 1:** Clinical and demographic characteristics of the study population

**Characteristic**	**Viable pregnancy**		**P value**
	**Yes (n = 40)**	**No (n = 65)**	
Age, years	36 (27–43)	36 (26–43)	0.97
BMI, kg/m2	22 (17–39)	21 (16–39)	0.26
Smoking	7 (17)	11 (17)	0.94
Arterial hypertension	0	1 (1)	0.43
Diabetes	0	0	-
History of cardio-cerebrovascular disease	1 (2)	0	0.20
History of venous thromboembolism	0	0	-
Indication for IVF			0.26
Male factor	11 (27)	26 (40)	
Tubaric	8 (20)	13 (20)	
Idiopathic	15 (37)	15 (23)	
Ovulatory	5 (12)	4 (6)	
Endometriosis	1 (2)	5 (8)	
Recurrent miscarriage	0	2 (3)	
Previous pregnancy	8 (20)	9 (14)	0.41
Previous miscarriage			0.62
No	37 (92)	57 (88)	
1	1 (2.5)	5 (8)	
2	1 (2.5)	2 (3)	
3	1 (2.5)	1 (1)	
≥ 3	0	0	
Number of previous cycles			0.31
0	32	30	
1	8	20	
2	3	3	
3	4	3	
>3	1	1	
Embryos transferred			0.98
1	1 (2)	2 (3)	
2	4 (10)	7 (11)	
3	34 (85)	55 (85)	
4	1 (2)	1 (1)	
Total dose of administered FSH (IU)	2775 (440–7157)	3225 (1000–6300)	0.13
Basal FSH mUI/ml	6.6 (4.2-12.3)	7.4 (2.1-18.3)	0.31

Legend: Continuous variables are presented as means ± standard deviations (SD), or as medians with their range, in case of skewed distributions. Data are presented as absolute numbers (%) for ordinal variables. *BMI* body-mass index; FSH = ; *IU* international units; *IVF* in-vitro fertilization; *r-hCG* recombinant human chorionic gonadotropin.

**Table 2:** Outcomes of the IVF procedure

	
GnRH antagonist protocol, n (%)	27/105 (26)
Retrieved oocytes, n (range)	7 (2–25)
Metaphase II oocytes, n (range)	5 (1–18)
Biochemical pregnancy only, n (%)	5/105 (5)
Implantation rate, % (range)	19% (0 to 100%)
Live birth rate, n (%)	32/105 (30)
Clinical pregnancy, n (%)	40/105 (38)
Twin pregnancy, n (%)	9/40 (22)
Triplets, n (%)	-
Ectopic pregnancy, n (%)	1/40 (2)
Pregnancy loss, n (%)	7/40 (17)

Legend: *GnRH* Gonadotropin Releasing Hormone.

Women achieving clinical pregnancy were similar for age, vascular risk factors, indication for IVF, and IVF procedures to women who had a pregnancy failure (Tables 1 and 2).

### D-dimer levels

**Figure 1:**
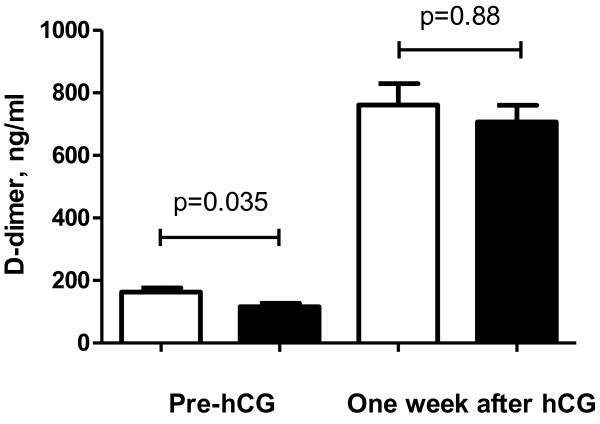
**D-dimer levels in women with and without a clinical pregnancy.** White bars: D-dimer in women without clinical pregnancy; grey bars: D-dimer in women with clinical pregnancy. r-hCG = Recombinant human chorionic gonadotropin.

Circulating levels of D-dimer measured just before the administration of r-hCG were significantly higher in women with a pregnancy failure (141 ng/mL, range 42 to 499 ng/mL) compared to those achieving a clinical pregnancy (115 ng/mL, range 16 to 215 ng/mL; p = 0.035) (Figure 1). This difference remained statistically significant after correction for age and indications for IVF in multivariable analysis (p = 0.032). D-dimer levels above the assay cut-off of positivity (200 ng/mL) were also associated with a worse outcome (p = 0.042).

One week after the administration of r-hCG, D-dimer values were significantly increased compared to the levels before r-hCG with no significant differences between women with and without a clinical pregnancy (748 ng/mL [range 161 to 1057 ng/mL] vs. 767 ng/mL [range 52 to 3140 ng/mL], p = 0.88).

## Discussion

In the current study we found that D-dimer levels measured before the administration of r-hCG were predictive of an unfavourable pregnancy outcome.

Relatively few studies with a low number of participants and with several methodological drawbacks explored the changes in haemostatic parameters during IVF [7-10]. In a small case–control study, Rogolino and colleagues found significantly higher concentrations of D-dimer, tissue factor, and protrombin fragments 1 + 2 in women hospitalised for OHSS relative to healthy controls [7]. Interestingly, the authors reported no differences between controls and women undergoing IVF without OHSS. In another series of 33 women, Curvers and colleagues found only modest changes in the plasma levels of natural anticoagulant proteins despite the considerable variation in hormonal concentrations during the IVF cycle [10].

The D-dimer test is a widely available test which is routinely used in the diagnostic work-up of venous thromboembolic events [11]. In patients at high risk of thrombotic complications, D-dimer measurement helps stratifying the risk of first or subsequent thrombosis and has the potential to identify subgroups warranting primary prophylaxis or prolonged anticoagulation [12,13]. To our knowledge, this is the first study to show a negative prognostic value of D-dimer in women undergoing IVF. These results extend previous observations which suggested an association between an unfavourable pregnancy outcome and D-dimer levels in women undergoing IVF and developing OHSS [7]. The present findings need to be considered cautiously due to the size of the included population which did not allow to explore the relationship of D-dimer with live birth rate or identify the D-dimer cut-off that best predict pregnancy success or failure. Future larger studies are warranted to clarify these issues.

D-dimer levels increased considerably one week after the trigger r-hCG injection, in agreement with earlier observations by Biron and colleagues who reported a significant activation of various haemostatic parameters following r-hCG [14]. While the mechanisms behind the activation of blood coagulation following r-hCG remain unclear, the changes in D-dimer concentrations observed in the current study were apparently not related to the final pregnancy outcome. Measurement of D-dimer at later time points after r-hCG could clarify whether these increased D-dimer levels persist long after the r-hCG stimulation or return to pre-stimulation values after a plateau phase. In this latter case, the high concentrations of D-dimer would reflect a transitory peak in blood coagulation activation not sufficiently prolonged to cause thrombosis of the maternal vessels.

In conclusion, we found that D-dimer concentrations seem to predict a higher risk of pregnancy failure in women undergoing IVF. If confirmed in future prospective studies, D-dimer could help identifying a group of patients who could benefit from prophylaxis to increase the pregnancy success rate.

## Competing interests

The authors declare that they have no competing interests.
